# Uncommon Vulvar Mass in a Neonate: A Case of Bartholin Gland Cyst

**DOI:** 10.7759/cureus.96004

**Published:** 2025-11-03

**Authors:** Kristin L Singer, Jason Skiwski

**Affiliations:** 1 Pediatrics, Lake Erie College of Osteopathic Medicine, Bradenton, USA; 2 Pediatrics, AdventHealth Gordon, Calhoun, USA

**Keywords:** bartholin gland abscess, bartholin gland cyst, interlabial mass, newborn vaginal mass, vaginal mass

## Abstract

Bartholin gland cysts are exceedingly rare in neonates and infants, with only four cases previously documented in the literature. We present the fifth reported case of a Bartholin gland abscess in a neonate. A term newborn presented with a 1-cm vaginal mass identified shortly after birth. An ultrasound of the mass was obtained and reported testicular-like tissue and the absence of a uterus, prompting an evaluation for disorders of sex development. Laboratory testing and karyotype were normal, and pediatric urology ultimately diagnosed and drained a Bartholin gland abscess, resulting in the resolution of the mass. This case highlights the importance of considering Bartholin gland cysts even in very young patients and emphasizes the need for careful interpretation of neonatal imaging, as misreading can lead to extensive diagnostic evaluations for a benign condition.

## Introduction

Bartholin gland cysts are rare in neonates, with only four reported cases in the literature. Although the cyst is benign, the differential diagnosis for a vaginal mass in this age group is broad and consists of conditions such as urethral prolapse, hymenal cyst, paraurethral gland cyst, bartholin gland cyst, complete or partial androgen insensitivity syndrome, and congenital adrenal hyperplasia [1]. The most common causes of a vaginal mass in neonates are hymenal and paraurethral cysts, but a mass in the vaginal opening could also represent ambiguous genitalia, prompting evaluation for disorders of sexual development. These masses may be differentiated from each other due to their size, appearance, and precise anatomic location [1].

Bartholin glands are pea-sized glands located posteriorly in the vaginal opening and are connected to ducts that drain mucus into the vaginal vestibule. When these ducts become blocked, the buildup of secretions results in the formation of a cyst or abscess. A Bartholin cyst is typically painless and asymptomatic, while an abscess often presents with surrounding cellulitis and lymph node enlargement [2]. Diagnosis of a Bartholin gland cyst is primarily clinical, with visualization of a cyst in the 4 or 8 o'clock position in the vaginal introitus being sufficient. Asymptomatic cysts may be observed for spontaneous resolution, while larger or painful cysts and abscesses may require incision and drainage or the often-preferred words catheter insertion, which is associated with lower recurrence rates [2].

Bartholin glands are typically not active until puberty, when vaginal lubrication begins to play a role in sexual and reproductive function. Bartholin cysts and abscesses most commonly affect women of reproductive age; however, they should still be considered in the differential diagnosis of a neonatal vaginal mass, despite the gland’s low level of activity at this stage. These cases may mimic other vulvovaginal pathologies and, in severe instances, cause urinary retention or obstructive uropathy, leading to hydronephrosis [1]. Recognition of this rare entity is therefore important to ensure appropriate management and to minimize unnecessary or stressful diagnostic investigations.

## Case presentation

**Figure 1:**
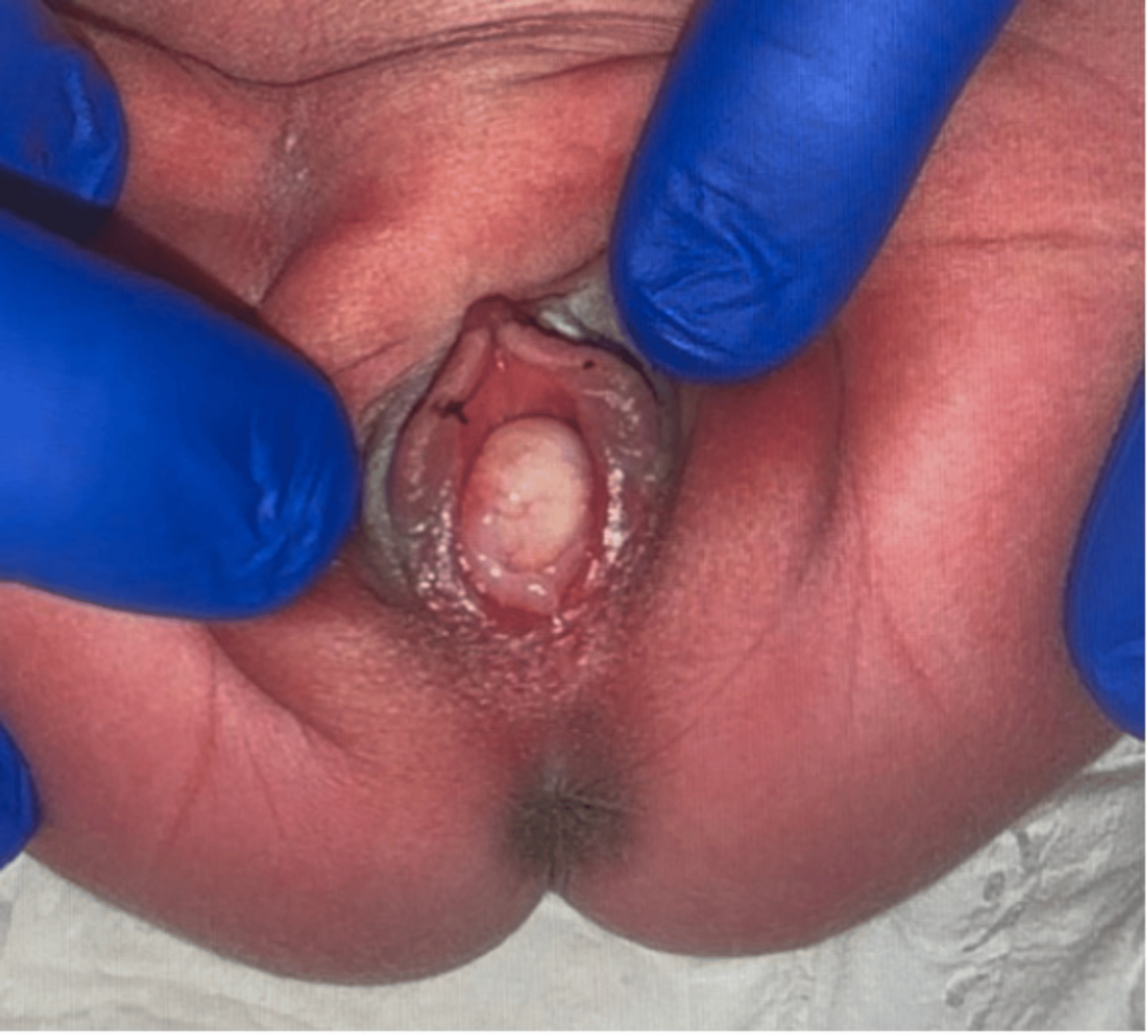
External genitalia showing an interlabial mass

**Figure 2:**
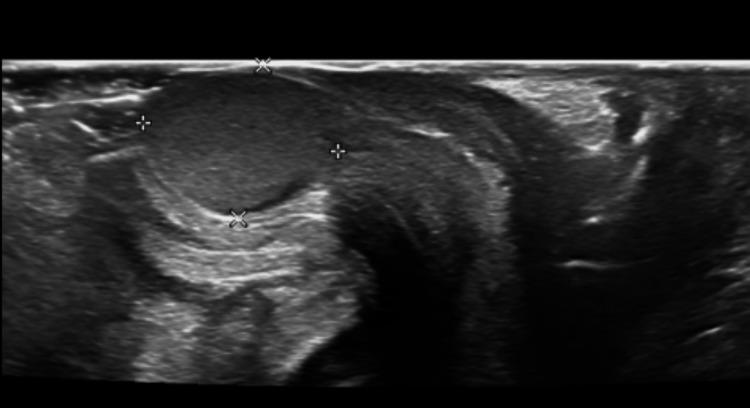
Sagittal translabial ultrasound view Sagittal translabial view showing the 1.1 x 1.0 x 1.0 cm mass in the vaginal canal. It is homogeneous and isoechoic. There is mild posterior acoustic enhancement. The presence of echogenicity and absence of an anechoic center made the lesion appear solid rather than cystic, which may have contributed to its misinterpretation as testicular tissue.

**Figure 3:**
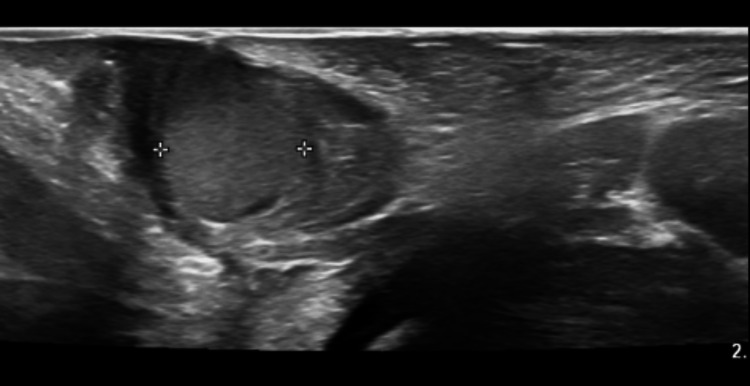
Transverse translabial ultrasound view Transverse translabial view showing the 1.1 x 1.0 x 1.0 cm mass in the vaginal canal. It is homogeneous and isoechoic. There is mild posterior acoustic enhancement. The presence of echogenicity and absence of an anechoic center made the lesion appear solid rather than cystic, which may have contributed to its misinterpretation as testicular tissue.

A neonate delivered at 39 weeks and 5 days to a Group B Streptococcus (GBS) positive mother was found to have a vaginal mass upon examination at 6 hours of life. The mass was round, moderately firm, and pearly pink in color, with visible vasculature, and measured to be about 1 cm in size (Figure 1). Birth history was significant for emergency cesarean section due to fetal intolerance of labor with bradycardia after failed labor induction for oligohydramnios. On initial newborn examination, the mass was thought to be a Bartholin gland cyst based on the location. Upon further inspection, the mass was suspected to be more of a vaginal protrusion rather than a labial mass. Due to this suspicion, imaging was ordered, and the differential was broadened. A pelvic ultrasound disclosed a circumscribed 1.1 x 1.0 x 1.0 cm homogenously isoechoic mass (Figures 2-3). In the imaging report, the mass was described as having a sonographic appearance similar to that of a testis, although the appearance was nonspecific. As seen on imaging, the mass is not purely anechoic, unlike a typical cyst. The ultrasound report identified a similar-appearing structure in the right hemipelvis, and no uterus was visualized. It was unclear whether there was a technical limitation to visualizing the uterus, given that it was a limited pelvic ultrasound. The final ultrasound impression stated that there was ambiguous internal genitourinary anatomy, and therefore, an MRI was recommended for further investigation of these sonographic findings. The family declined this recommendation. In light of these sonographic findings, disorders of sexual development were strongly considered in the differential diagnosis.

Due to the reported findings of a testicular-like structure and an absent uterus, a workup for androgen insensitivity syndrome (AIS) and congenital adrenal hyperplasia (CAH) was conducted. Karyotype analysis, testosterone, dihydrotestosterone, luteinizing hormone (LH), follicle-stimulating hormone (FSH), cortisol, 17-hydroxyprogesterone levels, and a complete metabolic panel (CMP) were ordered. The CMP results revealed normal sodium and potassium levels, which did not support the diagnosis of CAH. However, 17-hydroxyprogesterone levels were required to firmly rule out this diagnosis, which was within normal range at 48.26, so the patient could be safely discharged while awaiting karyotype results. Testosterone, dihydrotestosterone, LH, FSH, and cortisol levels were all within normal limits for age. After two weeks, the karyotype result revealed a normal female genotype of 46XX. This finding was unexpected, with the working diagnosis being AIS. During this time, prior to the karyotype results, the newborn was seen by her pediatrician and referred to pediatric urology. Pediatric urology described the mass as a 2-3 cm Bartholin cyst on the left side. Because the mass had increased in size compared to the initial presentation, it was decided to be drained in the office. The mass was gently opened using a 24-gauge needle, and fair, opaque fluid was expressed from the cyst, with minimal bleeding. Pressure was held, and the remaining fluid was expressed. Antibiotic ointment was applied to the vaginal introitus after drainage, and the cyst resolved. After review of the initial ultrasound, it was determined that the testicular-like appearance of the mass and the absence of a uterus were likely misinterpretations.

## Discussion

It was determined that the original, limited pelvic ultrasound misidentified the vaginal mass as testicular tissue. The absent uterus reported on ultrasound was later assumed to have just been missed on imaging.

Bartholin gland cysts are infrequently found in pre-pubertal girls, especially newborns. A large Bartholin gland cyst in a newborn can lead to complications, including urinary retention and infection [2]. In this case, the urethral meatus was nondisplaced and unobstructed by the cyst; the patient had voided within the first day of life.

Based on the US results, the initial working diagnoses were CAH and AIS. These were important to rule out, as they can have both physical and psychological consequences. CAH had to be ruled out immediately because of the life-threatening aspects of the disorder. CAH presents with early adrenal insufficiency with salt wasting and hypoglycemia, as well as skin hyperpigmentation and some degree of virilization of the external genitalia, regardless of sex chromosome. Adrenal insufficiency can rapidly progress to adrenal crisis, resulting in severe dehydration, hypotension, and shock, leading to death [3].

The revised differential diagnosis for an interlabial mass includes a Bartholin gland cyst, hymenal cyst, paraurethral cyst, imperforate hymen, urethral prolapse, rhabdomyosarcoma of the vagina or cervix, and urethral or vaginal polyps. The three vaginal cysts may appear strikingly similar on examination and can be differentiated by the presence or absence of urethral displacement [1].

Bartholin glands are remnants of the urogenital sinus and can therefore be associated with urinary tract malformations [3]. It has also been hypothesized that maternal estrogen can increase secretions, leading to ductal obstruction and cyst formation. This has been reported to occur in neonates with an imperforate hymen. The vaginal mucosa is stimulated by maternal estrogen, and the resulting secretions may form a hydrocolpos [1]. Further research is needed to establish a connection between maternal hormones and the development of Bartholin gland cysts in neonates. Another possible mechanism of bartholin gland cyst formation in neonates could be congenital narrowing of the duct [4].

Bartholin gland cysts are rare in neonates, with only four other cases having been reported in the literature [5-8]. In the first-ever reported case, a neonate born to a mother treated for Trichomonas vaginalis developed a labial abscess at day 3 of life. Incision and drainage were performed, and the infant was successfully treated with antibiotics [5]. The second case involves a one-month-old who presented with a four-day history of left labial swelling. Examination revealed a soft, tender, erythematous 2.2 × 1.3 cm cystic lesion that demonstrated hypo to anechoic content on ultrasound. Aspiration revealed pus, and outpatient incision and drainage resolved symptoms without recurrence [6]. The third reported case has a unique element in which the Bartholin cyst was also associated with a contralateral renal cyst and hydroureteronephrosis. The two-day-old neonate presented with a 5 cm Bartholin’s duct cyst and was unable to urinate. After unsuccessful urethral catheterization attempts, the cyst was resolved surgically under general anesthesia without recurrence [7]. The most recent case of a three-week-old term female presented with a tender, erythematous swelling of the left labia majora and inguinal lymphadenopathy. Ultrasound showed a 1.9 mL anechoic vulvar lesion. She was hospitalized and treated with amoxicillin; the abscess drained spontaneously after three days and did not recur [8].

As evidenced by these four prior cases, clinical diagnosis alone was uncommon, as perineal ultrasound was frequently utilized to characterize the lesion further and exclude other etiologies. Furthermore, although conservative management is typically recommended in adults, incision and drainage were performed in three of the four neonatal cases. In such instances, diagnostic imaging with ultrasound can aid in confirming the diagnosis or assessing masses that obstruct the urethral or vaginal orifices [9].

## Conclusions

In this case, a newborn girl presented with a vaginal mass initially raising concern for a disorder of sex development. Subsequent evaluation determined the lesion to be a Bartholin gland abscess, which was successfully treated with incision and drainage. This case highlights several important learning points in the evaluation of genital masses in newborns. Bartholin gland cysts or abscesses, although rare in newborns, should remain in the differential diagnosis of vaginal masses. Imaging is not required for diagnosis, as Bartholin gland abscesses can be diagnosed clinically. Imaging findings in neonates must be interpreted with caution, as small pelvic structures and variable anatomy can lead to misdiagnosis, such as mistaking a benign cyst for testicular tissue or failing to visualize a uterus. While the imaging findings in this case appropriately prompted a thorough workup, they highlight how the rarity of such conditions in newborns can lead to extensive diagnostic evaluations.
